# Posterior Tibial Plateau Offset Is Reduced During Total Knee Arthroplasty and Is Associated with Tibial Component Malpositioning

**DOI:** 10.3390/medsci14020192

**Published:** 2026-04-11

**Authors:** Luis V. Bürck, Rosa Berndt, Clemens Gwinner, Lorenz Pichler, Moses Kamal Dieter El Kayali

**Affiliations:** 1 Center for Musculoskeletal Surgery, Charité—Universitätsmedizin Berlin, 10117 Berlin, Germany; luis-vincent.buerck@charite.de (L.V.B.);; 2 Department of Orthopedics and Trauma-Surgery, Medical University of Vienna, 1090 Vienna, Austria; 3 Institute for Clinical and Experimental Surgery, Saarland University, 66421 Saarbrücken, Germany

**Keywords:** total knee arthroplasty, tibial component alignment, posterior tibial plateau offset, knee morphology, sagittal alignment

## Abstract

**Purpose:** The posterior tibial plateau offset (PTPO) is a parameter of sagittal plane bony tibia morphology with high variability and clinical relevance, particularly in cases involving stemmed tibial implants, where posterior tibial cortex interference may occur. However, its change during total knee arthroplasty (TKA), and its relationship to tibial component positioning remain unknown. **Methods:** Pre- and postoperative sagittal radiographs of 98 patients undergoing primary, mechanically aligned TKA using a single implant system were retrospectively analyzed. PTPO was measured as the distance between the tibial anatomical axis and the center of the tibial plateau or tibial component. Tibial component placement (TCP) was assessed anteriorly and posteriorly and categorized as anatomical (0–1 mm), mild (1–3 mm), or moderate (>3 mm) underhang (TCU) or overhang (TCO). Pre- and postoperative changes in PTPO were analyzed, preoperative PTPO was compared across TCP categories. Correlations with absolute anterior and posterior deviation from anatomical component placements were calculated. **Results:** PTPO showed high preoperative variability (mean 6.89 ± 3.69 mm) and was significantly reduced after TKA (5.89 ± 3.44 mm; mean change −1.06 ± 3.44 mm; *p* < 0.001). Higher preoperative PTPO was associated with anterior (*p* = 0.01) and posterior TCU (*p* = 0.02). PTPO showed a moderate correlation with anterior (r = 0.53, *p* < 0.01) and a strong correlation with posterior implant deviation (r = 0.68, *p* < 0.01). **Conclusions:** PTPO shows high variability among patients undergoing TKA, is significantly altered through surgery and correlates with tibial component malposition, particularly TCU. Surgeons should consider PTPO during preoperative planning to optimize tibial component positioning and reduce the risk of implant-to-bone conflict, especially when using stemmed implants. In patients with a high preoperative PTPO, accuracy-enhancing techniques such as computer navigation or robotic assistance may be considered.

## 1. Introduction

While the coronal alignment of the knee joint has received increasing attention in recent years, research on the sagittal alignment remains relatively scarce [1]. Investigations into parameters such as the posterior femoral condylar offset (PFCO) and the posterior tibial slope (PTS) have demonstrated the significant impact of sagittal plane alignment on knee function in total knee arthroplasty (TKA) [2,3,4,5,6,7]. However, to achieve a fully personalized approach to TKA through knee phenotyping, all aspects of bony morphology must be taken into account [8].

Among these, particular attention should be given to parameters exhibiting high inter-individual variability, such as the posterior tibial plateau offset (PTPO), which represents the offset of the tibial plateau relative to the tibial shaft in the sagittal plane [9]. First described by Qiheng et al. [9], PTPO was reported to average 7.23 mm with a standard deviation (SD) of 2.44 mm in healthy knees, and its variability has since been confirmed in a study of osteoarthritic knees as well [10]. The relevance of PTPO is further highlighted in cases requiring stemmed tibial implants, where interference between the implant stem and the posterior tibial cortex has been observed in up to 19% of cases which may contribute to component malposition or periprosthetic fractures [10].

Nevertheless, any parameter used for knee phenotyping should not only enable classification but also demonstrate clinical relevance. In this context, sagittal tibial component overhang and underhang, often resulting from a mismatch between implant size and the native anterior–posterior dimension of the tibial plateau, have both been associated with inferior outcomes following TKA. Tibial component overhang (TCO) can lead to soft tissue impingement, particularly involving the popliteus tendon, contributing to persistent postoperative pain and reduced patient satisfaction [11]. In contrast, tibial component underhang (TCU) may result in insufficient bone coverage, potentially promoting tibial bone resorption and increasing the risk of implant loosening and early failure [12,13]. Taken together, achieving optimal tibial component positioning remains challenging in clinical practice.

Given that sagittal tibial morphology directly influences the anterior–posterior dimensions and contour of the proximal tibia, parameters such as PTPO may affect the ability to achieve anatomical implant coverage. However, evidence directly linking PTPO to implant sizing or component positioning remains lacking. Furthermore, it remains unclear to what extent PTPO is altered by the tibial cut during TKA, and to what extent its variability influences tibial component fit.

Therefore, the aim of this study was to evaluate PTPO in patients undergoing non-robotic-assisted mechanically aligned TKA and to assess its potential impact on anterior–posterior tibial component placement (TCP). The hypotheses tested were: (1) PTPO is significantly changed through TKA and (2) preoperative PTPO is associated with tibial component malpositioning.

## 2. Methods

### 2.1. Study Design

This retrospective cohort study was conducted at a single academic orthopedic center to evaluate changes in PTPO following TKA and to assess its association with tibial component positioning.

### 2.2. Patients

**Figure 1:**
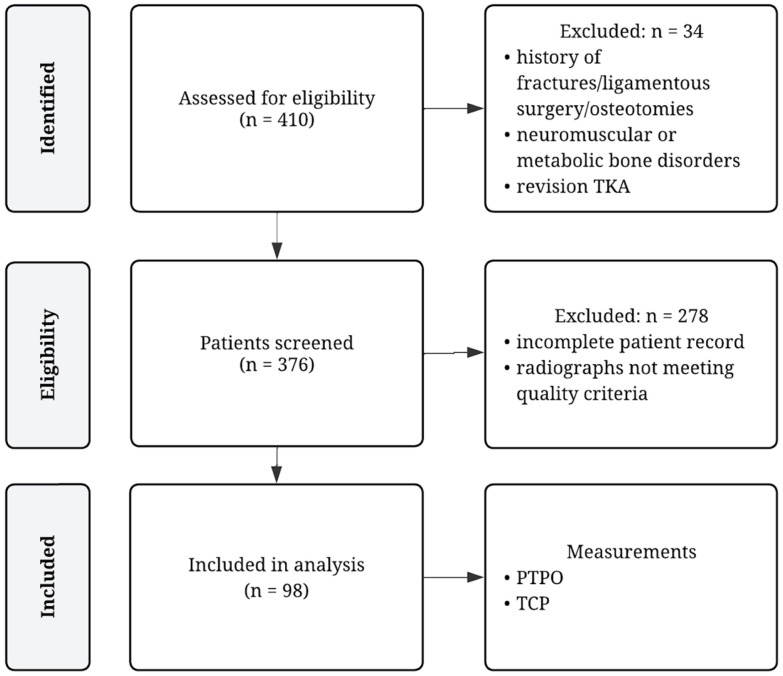
Flow chart of patient selection. PTPO = posterior tibial plateau offset; TCP = tibial component placement; TKA = total knee arthroplasty.

A total of 410 consecutive patients undergoing conventional TKA at our academic orthopedic surgery center by multiple surgeons using one total knee system between October 2020 and March 2023 were screened for inclusion. Inclusion criteria were defined as follows: patients undergoing primary TKA of either knee, availability of pre- and postoperative knee radiographs, completeness of patient records, and patient consent. Exclusion criteria were history of fractures or ligamentous injury, previous ligamentous surgery or osteotomies of the operated knee, unicompartmental or revision knee arthroplasty, known neuromuscular or metabolic bone disorders, insufficient patient record and radiographs not meeting our quality criteria as described under ‘Radiological assessment’. The process of patient selection is illustrated in Figure 1.

Demographic data (age, sex, BMI), surgical data (date of surgery, surgical technique, implant type) and pre- and postoperative knee radiographs were collected. Patient demographics and surgical data are presented in Table 1.

### 2.3. Surgical Technique

TKA was performed by eleven surgeons using conventional mechanical alignment strategy with a standard medial approach to the knee and a tourniquet [14,15]. Only one knee system (Attune, DePuy Synthes, Raynham, MA, USA) with either cruciate-retaining (CR) or posterior-stabilized (PS) design was used. Surgical technique guidelines by the manufacturer were followed strictly. Implant fixation was achieved by cementation or press-fit.

### 2.4. Radiological Assessment

Preoperative radiographs were obtained at the time of indication for TKA, and postoperative radiographs were taken during the early postoperative period, within 14 days after surgery. For standardization purposes flexion in the knee joint, parallel positioning to the sagittal plane of the detector and center of radiograph at the patellofemoral joint line was aimed for. All radiographs were calibrated using a standard radiographic reference ball of 25.4 mm (1 inch) in diameter. Cases with excessive knee rotation and/or abduction/adduction, as well as those with insufficient tibial shaft visualization (<15 cm), were excluded, in line with previous investigations demonstrating that malpositioning on lateral radiographs can compromise the accuracy of sagittal bony measurements such as the PTS [6,16].

**Figure 2:**
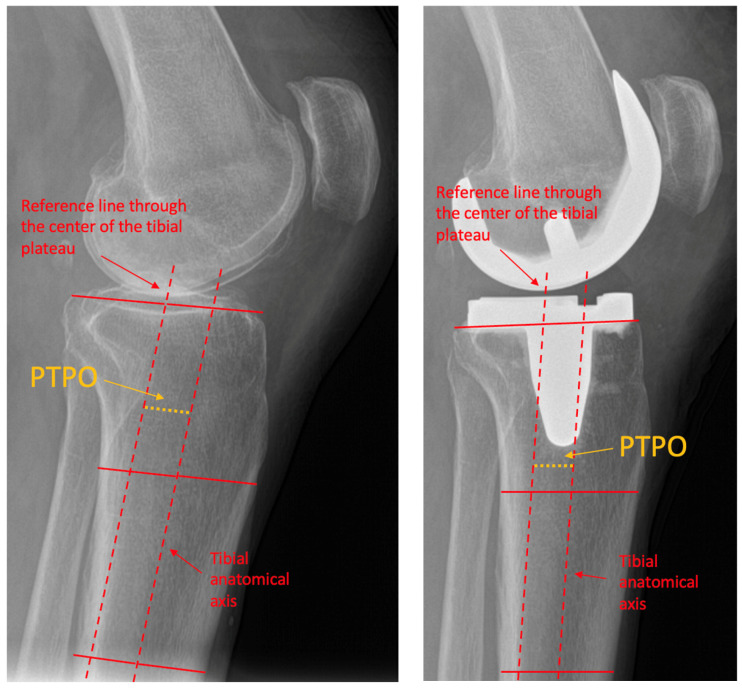
Measurement of PTPO. (**Left**) lateral preoperative knee radiograph showcasing PTPO measurement. (**Right**) lateral postoperative knee radiograph showcasing PTPO measurement. PTPO = posterior tibial plateau offset.

According to previous studies PTPO was defined as the offset of the tibial plateau in relation to the tibial anatomical axis [9,10]. For its measurement the tibial anatomical axis was defined by identifying two mid-diaphyseal points, one proximally and one distally, and connecting them with a straight reference line. A second line was drawn at the joint level: preoperatively through the center of the tibial plateau, and postoperatively through the center of the tibial component. The perpendicular distance between centers of both lines in millimeters was reported as the PTPO. The measurement technique is presented in Figure 2.

**Figure 3:**
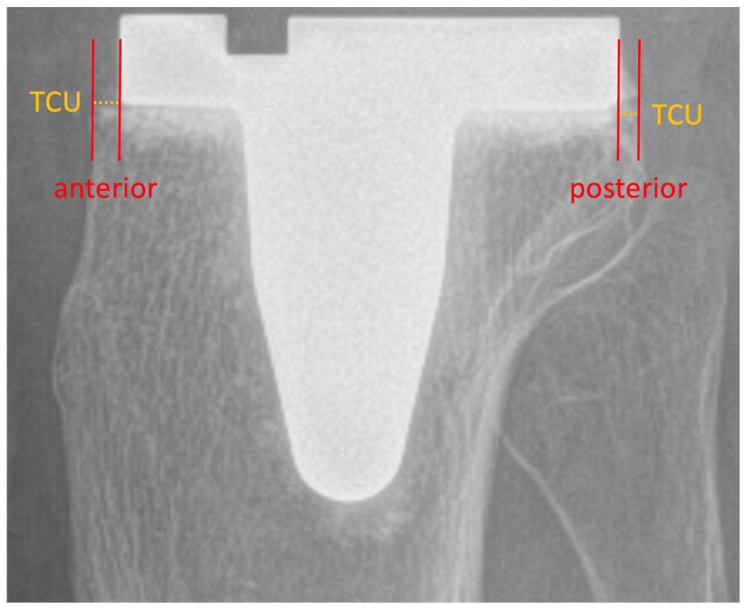
Measurement of TCP. Lateral postoperative knee radiograph showcasing measurement of anterior and posterior tibial component placement. TCP = tibial component placement. TCU = tibial component underhang.

Tibial component placement was measured on lateral knee radiographs at two points, anterior and posterior, respectively, as described in previous studies [17,18]. As showcased in Figure 3, the distance between a tangent line of the tibial baseplate and the edge of the ipsilateral tibial cutting surface was measured. TCO was defined as the tibial component extending beyond the tibial plateau at either position, while TCU was defined as incomplete coverage of the tibial plateau by the component.

Osteophytes, defined as bony projections extending beyond the normal cortical margins at the joint surface, were excluded from all PTPO and TCP measurements to ensure accurate representation of the native bony anatomy.

Two independent observers, blinded to patient clinical data, performed the measurements of the PTPO and the TCP. Intraclass correlation coefficients (ICCs) demonstrated excellent interobserver reliability for both preoperative (PTPO: ICC, 0.91) and postoperative measurements (PTPO: ICC, 0.92; TCP: ICC, 0.81) [19]. The mean values calculated between raters were used for further analysis.

Measurements were performed out using a PACS workstation (Centricity RIS-I 4.2. Plus, GE Healthcare, Milwaukee, WI, USA). Radiographs were taken using a digital radiography system (XGEO GC85A, Samsung, Seoul, Republic of Korea).

### 2.5. Statistical Analysis

The Shapiro–Wilk test was used to assess normality of data distribution. Descriptive parameters including mean, SD, median, and range were calculated where appropriate. For normally distributed variables, two-sided *t*-tests were performed; otherwise, the Wilcoxon signed-rank test was used. The *p*-value (*p*) was reported for each test of significance. Correlation between variables were assessed using Pearson’s correlation coefficient for normally distributed data, and Spearman’s rank correlation coefficient for non-normally distributed data. For each correlation, the correlation coefficient (r) and corresponding *p*-value were reported.

Pre- to postoperative differences in PTPO were assessed for statistical significance using a two-sided paired *t*-test. Preoperative PTPO values were compared between female and male patients using a two-sided independent *t*-test. Based on previous studies, TCP was graded as follows: anatomical positioning (0 to 1 mm TCO or TCU); mild malposition (1 to 3 mm), and moderate malposition (>3 mm TCO or TCU) [18,20,21]. Preoperative PTPO was compared between knees with anatomical tibial component positioning and those with any degree of TCO or TCU (mild or moderate, anterior or posterior) using a two-sided independent *t*-test.

Additionally, to evaluate the relationship between preoperative PTPO and the magnitude of tibial component malposition, absolute values of anterior and posterior tibial component deviation (the absolute distance from anatomical placement in mm) were calculated and reported. Correlation between preoperative PTPO and absolute anterior and posterior deviation was assessed using Pearson’s correlation coefficient.

A significance level of *p* < 0.05 was considered statistically significant. A correlation coefficient (r) of 0–0.19 was regarded as very weak, 0.2–0.39 as weak, 0.40–0.59 as moderate, 0.6–0.79 as strong, and 0.8–1 as very strong correlation [22].

A post hoc power analysis was performed to determine the statistical power achieved with the given sample size. Power was calculated to be 0.93 (two-tailed *t*-test; calculated effect size based on pre- and postoperative measurements: 0.35, α error probability 0.05, using G*Power software, version 3.1.9.6) [23].

All calculations were performed using Microsoft Excel for macOS (Version 2024, Microsoft, Redmond, WA, USA) and SPSS (IBM Corp. Released 2022. IBM SPSS Statistics for macOS, Version 29.0.0.0 Armonk, NY, USA).

### 2.6. Ethical Aspects

The study protocol was approved by the Ethikkommission Charité (protocol code EA2/016/21) on 5 March 2021 and was conducted in accordance with the Declaration of Helsinki. Written informed consent was obtained from all patients.

## 3. Results

A total of 98 (53 [54%] females and 45 [46%] males) consecutive patients met the inclusion criteria.

The mean preoperative PTPO was 6.89 mm ± 3.69 mm and was significantly reduced postoperatively to 5.89 mm ± 3.44 mm. The mean change in PTPO was −1.06 mm ± 3.44 mm (*p* < 0.01). There was no statistically significant difference in preoperative PTPO between females (6.78 ± 3.58) and males (7.02 ± 3.82 mm; *p* = 0.68).

Anatomical tibial component positioning was found in 20 cases (20.4%) anteriorly, and in 12 cases (12.2%) posteriorly. Anterior and posterior TCO was observed in 6 (6.1%) and 9 (9.2%) cases, respectively. Anterior TCU was present in 72 cases (73.5%), and posterior TCU in 77 cases (78.6%).

Mean preoperative PTPO was significantly higher in cases with anterior TCU compared to anatomically positioned components (7.93 ± 5.45 mm vs. 4.79 ± 3.21 mm; *p* = 0.01). Similarly, in cases with posterior TCU, mean preoperative PTPO was significantly higher than in anatomically placed components (9.67 ± 4.10 mm vs. 4.25 ± 2.45 mm; *p* = 0.02).

The full distribution of cases by anterior/posterior TCP, severity of TCU/TCO, sex, and corresponding preoperative PTPO values with *p*-values is provided in Table 2.

The mean absolute anterior tibial component deviation was 2.59 ± 1.32 mm, and the mean absolute posterior deviation was 2.98 ± 1.41 mm. A moderate positive correlation was found between preoperative PTPO and anterior deviation (*r* = 0.53, *p* < 0.01), while a strong correlation was observed with posterior deviation (*r* = 0.68, *p* < 0.01).

## 4. Discussion

The most important finding of our study is that PTPO was significantly reduced through TKA, confirming our first hypothesis. Furthermore, as assumed by our second hypothesis, cases with anterior and posterior TCU exhibited significantly higher preoperative PTPO values compared to the anatomically placed group. In addition, a moderate to strong positive correlation was observed between preoperative PTPO and absolute deviation from anatomical tibial component positioning, suggesting that greater preoperative PTPO is associated with tibial component malposition in the sagittal plane.

Recent developments in TKA promise improved patient outcomes and satisfaction through more personalized surgical techniques and implant designs [24,25]. This shift away from classical mechanical alignment (MA) principles [26] toward phenotype-based approaches has highlighted the importance of understanding individual anatomical variations [27,28].

Traditionally, the MA approach aimed to achieve a neutral hip-knee-ankle (HKA) angle [29], often resulting in alterations of the overall three-dimensional (3D) alignment of the knee joint [30,31]. This is reflected in our findings, as the significant pre- to postoperative reduction in PTPO indicates a modification of the native bony anatomy and may be associated with changes in the sagittal plane knee kinematics. While the clinical and kinematic relevance of PTPO variability remains largely unexplored, other sagittal parameters of the knee joint have been well studied. The PTS directly influences knee kinematics by altering the flexion gap and thereby affecting postoperative range of motion [32,33,34]. Similarly, changes in anterior femoral condylar offset can lead to overstuffing or understuffing of the patellofemoral joint, which is a recognized source of anterior knee pain and cause of revision surgery [35]. Increasing posterior femoral condylar offset can result in excessive flexion gap tightness and flexion–extension mismatch [5,36].

Our results contribute to the limited body of literature on PTPO by confirming the relatively high variability in proximal morphology, as reflected by a mean preoperative PTPO of 6.89 ± 3.69 mm in our cohort, highlighting considerable inter-individual differences [9,10]. Interestingly, we found no statistically significant difference in PTPO between female and male patients, a finding that aligns with previous observations for the PTS [37,38,39,40,41]. In contrast, sex-specific differences have been reported for the PFCO [42].

Sex-specific considerations are increasingly recognized as important in orthopedic research and clinical practice, as biological and social factors influence musculoskeletal conditions and treatment response [43]. In the context of TKA, several studies have reported higher rates of femoral and tibial component malpositioning in female patients [44,45,46].

TCU and TCU are surprisingly common, with reported rates of up to 80% [12,18,44,47,48], which was confirmed in our cohort, where only 32% of cases showed an anatomical tibial component fit. Achieving anatomical implant positioning is clinically relevant, as undersizing is associated with bone resorption, implant migration, and early failure, whereas oversizing can lead to soft tissue impingement, persistent pain, and inferior function outcomes [18,45,47,48,49,50].

Identified risk factors for non-anatomical tibial component placement include implant design, female sex, age and asymmetry of the tibial plateau [46,47]. In our study, both anterior and posterior TCU were significantly more common in cases with higher preoperative PTPO, and greater PTPO was moderately to strongly correlated with increased deviation from anatomical tibial component positioning. Thus, our results suggest that preoperative PTPO may represent an additional risk factor for tibial component malpositioning. In cases with high PTPO, achieving anatomical fit maybe be more challenging, potentially due to limited compatibility of standard implant geometries with posteriorly extended tibial morphology. When faced with this mismatch intraoperatively, surgeons may be more inclined to accept minor TCU in order to avoid TCO and its associated risk of soft tissue impingement.

**Figure 4:**
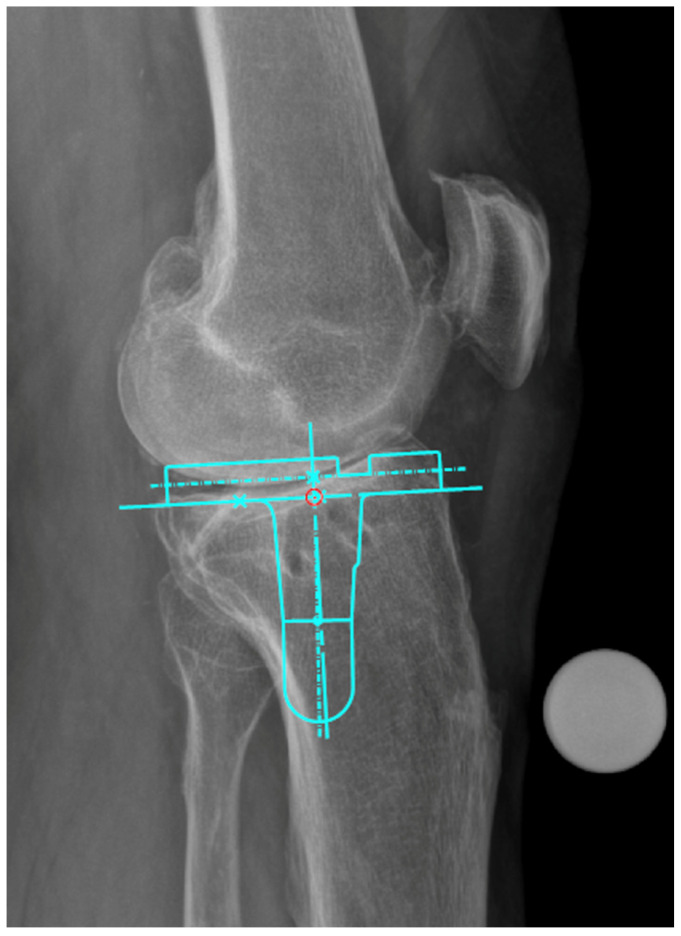
Implant-to-bone conflict with a stemmed tibial implant in a patient with high posterior tibial plateau offset. Lateral preoperative knee radiograph with digital templating of a stemmed tibial component using TraumaCad (Version 2.5; Brainlab Ltd., Munich, Germany).

Therefore, surgeons should be aware of PTPO variability and consider it during preoperative planning, particularly in cases where standard implant designs may not accommodate the patient’s sagittal tibial morphology, and in cases where stemmed implants are used, as previously reported by Secrist et al. [10]. A practical illustration of this relationship is shown in Figure 4, which depicts a lateral radiograph of a knee with high PTPO, where the templated stemmed tibial component impinges on the posterior tibial cortex when aiming for anatomical component positioning. This highlights the relevance of sagittal tibial morphology and its variability and emphasizes the need for implant designs that more reliably accommodate individual anatomical differences.

Diligent and individualized preoperative planning should be emphasized [51,52]. On the other hand, in the age of personalized medicine implant designs could better account for variability in patients’ morphology. More adaptable tibial baseplates may help reduce component malpositioning, and manufactures should be encouraged to further explore solutions that accommodate this variability.

This study carries some limitations beyond those inherent to its retrospective design. The analysis relied entirely on two-dimensional radiographic images. This approach does not fully capture the complex 3D geometry of the proximal tibia, including medial–lateral asymmetry and variations in plateau morphology. As a result, PTPO measurements derived from plain radiographs may oversimplify the underlying anatomy and may be influenced by projectional factors. Advanced imaging modalities such as computed tomography could provide a more comprehensive and anatomically accurate assessment of PTPO and TCP. In particular, 3D analysis may allow separate evaluation of medial and lateral plateau morphology and improve the understanding of how sagittal offset relates to implant positioning. Consequently, the relationships observed in this study may differ when assessed using 3D imaging, and future studies should incorporate such techniques to validate and extend our findings. Moreover, all patients were treated with MA TKA; future research should examine whether personalized alignment strategies influence PTPO and TCP differently. The patient population included in this study was drawn from a single institution with limited ethnic diversity and a predominance of Caucasian individuals, thereby limiting the generalizability of findings to other populations. The use of only one TKA system restricts the applicability of our results to other implant designs; however, it also minimizes variability in surgical technique and instrumentation as potential confounding factors. Furthermore, the absence of both patient-reported outcome measures, objective functional assessments, and revision-related outcomes represents an important limitation of this study. As a result, our findings do not allow conclusions as to whether PTPO is merely a radiographic descriptor of sagittal tibial morphology or a true determinant of postoperative function, symptoms, implant survival, or the risk of revision after TKA. Accordingly, the clinical relevance of the observed postoperative reduction in PTPO and its association with TCP remains uncertain and should be specifically addressed in future studies combining radiographic, functional, and longitudinal outcome data. Interpretation of significance tests should also be approached with caution due to the relatively small number of cases in some categories, particularly in the comparison of mean PTPO values between anatomically placed components and those with TCU or TCO. Nevertheless, these findings are supported by the results of the correlation analysis, which demonstrated consistent associations between PTPO and TCP.

A truly personalized approach in TKA should consider not only coronal alignment but also all other planes of the knee joint. Moreover, it may need to go beyond static morphology to include dynamic changes during various phases of gait and loading in the lower limb [53,54]. Therefore, the functional impact of PTPO on knee kinematics and (long-term) clinical outcomes should be addressed in future studies.

## 5. Conclusions

This study confirms the high variability of preoperative PTPO in patients undergoing TKA and demonstrates a significant reduction following mechanically aligned TKA. Higher preoperative PTPO values were associated with greater deviation from anatomical TCP, particularly TCU. These findings should be interpreted within the context of mechanically aligned TKA using a single implant system. Future studies should further investigate the relationship between PTPO, sagittal alignment parameters, and their impact on knee kinematics and clinical outcomes in TKA.

## Figures and Tables

**Table 1:** Patient demographics and surgical data.

	Male	Female	Overall
Cases, n	45 (45.92%)	53 (54.08%)	98 (100%)
Age, years	70.69 (range; 44.23 to 90.44)	74.74 (range; 54.31 to 89.82)	72.88 (range; 44.23 to 90.44)
BMI	29.56 (range; 19.30 to 44.90)	29.15 (range; 19.30 to 44.90)	28.43 (range; 20.70 to 43.20)
*Implant type*			
Cruciate retaining	38 (38.78%)	39 (39.80%)	77 (78.57%)
Posterior stabilized	11 (11.22%)	10 (10.20%)	21 (21.43%)
*Surgical technique*			
Cemented	41 (41.84%)	43 (43.88%)	84 (85.71%)
Cementless	8 (8.16%)	6 (6.12%)	14 (14.29%)

Values presented represent means if not stated otherwise; BMI = body mass index.

**Table 2:** Tibial component positioning and posterior tibial plateau offset.

TCP	Cases with Anatomical Positioning (0–1 mm)	Cases with Any TCO	Cases with Mild TCO (1–3 mm)	Cases with Moderate TCO (>3 mm)	*p*-Value	Cases with Any TCU	Cases with Mild TCU (1–3 mm)	Cases with Moderate TCU (>3 mm)	*p*-Value
anterior	20 (20.41%)	6 (6.12%)	6 (6.12%)	n.a.		72 (73.47%)	41 (41.84%)	31 (31.63%)	
males		4 (4.08%)				36 (36.73%)			
females		2 (2.04%)				36 (36.73%)			
posterior	12 (12.24%)	9 (9.18%)	8 (8.16%)	1 (1.02%)		77 (78.57%)	34 (34.69%)	43 (43.88%)	
males		4 (4.08%)				34 (34,69%)			
females		5 (5.10%)				43 (43.88%)			
PTPO									
anterior	4.79 ± 3.21 mm	6.03 ± 2.35 mm			>0.05	7.93 ± 5.45 mm			0.01
posterior	4.25 ± 2.45 mm	5.03 ± 3.01 mm			>0.05	9.67 ± 4.10 mm			0.02

TCP = Tibial component placement; TCO = Tibial component overhang; TCU = Tibial component underhang; PTPO = Posterior Tibial Plateau Offset.

## Data Availability

The data presented in this study are available upon reasonable request from the corresponding author due to ethical and data protection restrictions involving patient information.

## Abbreviations

3D	Three-dimensional
BMI	Body mass index
CR	Cruciate-retaining
HKA	Hip-knee-ankle angle
ICC	Intraclass correlation coefficient
MA	Mechanical Alignment
PFCO	Posterior femoral condylar offset
PS	Posterior-stabilized
PTPO	Posterior tibial plateau offset
PTS	Posterior tibial slope
SD	Standard deviation
TCO	Tibial component overhang
TCP	Tibial component placement
TCU	Tibial component underhang
TKA	Total knee arthroplasty
WA	Washington
WI	Wisconsin
